# Increase of vancomycin-resistant *Enterococcus faecium* strain type ST117 CT71 at Charité - Universitätsmedizin Berlin, 2008 to 2018

**DOI:** 10.1186/s13756-020-00754-1

**Published:** 2020-07-16

**Authors:** Anna Weber, Friederike Maechler, Frank Schwab, Petra Gastmeier, Axel Kola

**Affiliations:** Institute of Hygiene and Environmental Medicine, Charité – Universitätsmedizin Berlin, Hindenburgdamm 27, 12203 Berlin, Germany

**Keywords:** Vancomycin-resistant *Enterococcus faecium*, Whole genome sequencing, ST117, CT71, Virulence factors, Resistance genes

## Abstract

**Background:**

In addition to an overall rise in vancomycin-resistant *Enterococcus faecium* (VREfm), an increase in certain strain types marked by sequence type (ST) and cluster type (CT) has been reported in Germany over the past few years. Outbreak analyses at Charité - Universitätsmedizin Berlin revealed the frequent occurrence of VREfm ST117 CT71 isolates in 2017 and 2018. To investigate whether ST117 CT71 have emerged in recent years or whether these strains have been circulating for a longer time, we retrospectively analyzed non-outbreak strains that occurred between 2008 and 2018 to identify frequent STs and CTs.

**Methods:**

In total, 120 VREfm isolates obtained from clinical and surveillance cultures from the years 2008, 2013, 2015, and 2018 were analyzed. Thirty isolates per year comprising the first 7–8 non-outbreak isolates of each quarter of the respective year were sequenced using whole genome sequencing. MLST and cgMLST were determined as well as resistance genes and virulence factors. Risk factors for VREfm ST117 were analyzed in a multivariable analysis with patient characteristics as possible confounders.

**Results:**

The percentage of VREfm of type ST117 increased from 17% in 2008 to 57% in 2018 (*p* = 0.012). In 2008, *vanA* genotype accounted for 80% of all ST117 isolates compared to 6% in 2018. *VanB* CT71 first appeared in 2018 and predominated over all other ST117 at 43% (*p* < 0.0001).

The set of resistance genes (*msrC*, *efmA, erm(B)*, *dfrG*, *aac(6′)-Ii, gyrA, parC* and *pbp5)* and virulence factors (*acm*, *esp*, *hylEfm*, *ecbA* and *sgrA*) in CT71 was also found in other ST117 non-CT71 strains, mainly in CT36. The study population did not differ among the different calendar years analyzed in terms of age, gender, length of stay, or ward type (each *p* > 0.2).

**Conclusion:**

This study revealed an increase in ST117 strains from 2008 to 2018, accompanied by a shift toward CT71 strains with the *vanB* genotype in 2018. We did not detect resistance or virulence traits in CT71 that could confer survival advantage compared to other CTs among ST117 strains. To date, it is not clear why ST117 and in particular strain type ST117 CT71 predominates over other strains.

## Background

In recent years, Vancomycin-resistant enterococci (VRE) have been on the rise among hospitalized patients in Germany [1]. Infections with VRE may result in an increased length of stay, higher mortality, and greater costs of hospitalization [2]. Risk factors for colonization or infection by VRE include long periods of hospitalization, increased antibiotic consumption, co-morbidities, immunosuppression, and exposure to patients colonized or infected with VRE [3]. Patients colonized with VRE and the patient environment may represent reservoirs for transmission because of the tenacity of VRE and its long survival time on dry surfaces [3, 4]. Vancomycin resistance is mediated through different genotypes of a gene cluster, *vanA* to *vanN*, which are located on plasmids or in the chromosome [5, 6]. Vancomycin-resistant *Enterococcus faecium* (VREfm) is able to acquire plasmids and insertion elements rapidly. Consequently a variety of resistance and virulence genotypes have emerged [7].

In recent years, the German National Reference Centre for Staphylococci and Enterococci has reported the frequent occurrence of VREfm strains of sequence type ST117 as determined by multi-locus sequence typing (MLST). Of their collection of 91 isolates from blood samples in 2016, more than half were ST117 [8]. Further analysis of ST was based on core genome multi-locus sequence typing (cgMLST), which identified frequent subclusters CT71 and CT36 [8].

Unpublished outbreak analyses at Charité - Universitätsmedizin Berlin (Charité) also revealed the frequent occurrence of VREfm ST117 CT71 isolates in 2017 and 2018. In order to understand the local epidemiology and strain characteristics, we investigated whether ST117 (and more specifically ST117 CT71) has only emerged in recent years or whether these strains have instead been circulating for a longer time and have been identified more often through the broad use of molecular typing methods.

Thus, we retrospectively analyzed trends in ST and CT types among non-outbreak strains in the past decade.

## Methods

### Study population

We retrospectively analyzed VREfm cultures, clinical and surveillance, routinely collected at Charité at five-year intervals in 2008, 2013, and 2018. The first 7–8 consecutive non-outbreak isolates per quarter from all Charité wards were included, comprising VRE positive clinical or screening cultures from individual patients. Because a recent publication suggested an increase in CT71 in Germany between 2015 and 2016 [8], isolates collected in 2015 were included according to the protocol mentioned above, resulting in 30 isolates per year and a total number of 120 isolates. The Charité is a 3000 bed, acute care facility and has three hospital sites located in three different districts of the city. The isolates in this study were obtained from all three sites. Charité screening protocols did not change over the study period and required surveillance cultures from previously known VRE carriers, all patients admitted to hematology/oncology wards, and patients that shared a room with a VRE carrier.

We retrieved epidemiological data from the patient data management system and included patient age and sex, date of sampling, ward, length of stay (LOS) at the time of specimen collection, and specimen collection site.

In our analysis, we included both clinical and screening cultures. Due to Data Protection Regulations, we lack the necessary data to diagnose associated infections. VREfm samples comprised rectal swabs (*n* = 76, 63%), blood cultures (n = 7, 6%), urine samples (*n* = 15, 13%), wound swabs (*n* = 5, 4%), stool samples (*n* = 9, 8%), nasal- and throat swabs (*n* = 2, 2%), and other clinical cultures (n = 5, 4%). Rectal swabs, throat swabs, and nasal swabs were considered screening specimens, all others were considered clinical specimens. Wards were divided into four categories: intensive care unit (ICU), hematology/oncology, surgery, and others. The latter category included standard care wards such as nephrology, cardiology and gastroenterology. Multiple assignment of one sample to different categories of ward type was not allowed.

Determination of VRE was performed using chromID® VRE agar plates (bioMérieux, Marcy-l’Étoile, France) and disc diffusion method with 5 μg vancomycin and 30 μg teicoplanin from MASTDISCS® (Mast Group Ltd., Bottle, United Kingdom) as recommended by the European Committee on Antimicrobial Susceptibility Testing (EUCAST). In addition, we tested for the presence of *van* genes (*vanA*, *vanB* and *vanC*) using multiplex PCR according to the study of Patel et al. [9]. Since 2017, we have mainly been using the Amplex assay eazyplex® VRE (Amplex Biosystems GmbH, Giessen, Germany) for the detection of *vanA* and *vanB*. Vitek®2 System (bioMérieux, Marcy-l’Étoile, France) or MALDI-TOF MS (Bruker Daltonics, Bremen, Germany) were used for identification and antimicrobial susceptibility testing since 2013. Moreover, we performed an alcohol tolerance assay as described in Pidot et al. [10] for a specific set of strains collected in this study against two different concentrations of isopropanol (23 and 60%).

### Whole genome sequencing and bioinformatic analyses

VREfm isolates were stored as cryocultures, subcultured on blood agar and incubated overnight at 37 °C. DNA extraction was performed using the UltraClean Microbial DNA isolation kit following the manufacturer’s instructions (Qiagen, Hilden, Germany). The quantity and purity of the DNA was measured by QuantiFluor ONE dsDNA System (Promega GmbH, Mannheim, Germany) and Eppendorf Biophotometer (Eppendorf AG, Hamburg, Germany). Short read sequencing libraries were generated from genomic DNA using the Nextera XT DNA library preparation kit (Illumina Inc., San Diego, USA) and were sequenced on the MiSeq system (Illumina Inc., San Diego, USA) with 250-cycle paired-end chemistry according to the manufacturer’s instructions. Isolates were sequenced to reach 100-fold coverage. After sequencing quality-trimming, de novo assembly with the Velvet assembler and gene-by-gene comparison approach using the SeqSphere+ software version 4.1.9 (Ridom GmbH, Muenster, Germany) were performed. For the gene-by-gene comparison, the *E.faecium* cgMLST task template with default parameters (suggested threshold: ≤ 20 alleles difference) and *E.faecium* reference genome NC_017022.1 (GenBank accession number: GCA_000250945.1) was used to extract MLST and cgMLST data as described previously [11]. The quality of the samples and sequencing runs was checked using Fast QC (https://github.com/s-andrews/FastQC), and Illumina Analysis Viewer (http://emea.support.illumina.com/sequencing/sequencing_software/sequencing_analysis_viewer_sav/downloads.html). The ResFinder and VirulenceFinder web server (http://www.genomicepidemiology.org) was used to identify resistance genes and virulence factors, using ResFinder3.2 with the setting searching for acquired antimicrobial resistance genes as well as chromosomal mutations associated with antibiotic resistance, and VirulenceFinder2.0 (respectively threshold of 90% minimum sequence identity and 60% minimum length identity cut-off).

In parallel, the recently published ASA^3^P analysis pipeline was used [12]. Briefly, raw sequencing reads were quality clipped with Trimmomatic [13] and de novo assembled with SPAdes [14]. Contigs were rearranged using MeDuSa [15] and annotated with Prokka [16]. The ST clade was determined using BLAST+ and the PubMLST.org database. Antibiotic resistance genes and virulence factors were analyzed with the use of CARD database [17] and VFDB [18]. Additionally, a phylogenetic approximately maximum-likelihood tree via FastTreeMP [19] was created. The visualization of the phylogenetic tree was performed using Microreact [20]. The Prokka-annotated coding sequences of each genome were also used as input for Roary v3.12.0 with default settings to perform a pangenome analysis [21]. The results were visualized using the roary_plots python script. The “Core” pangenome contains all genes common to every isolate while the “Accessory” pangenome contains all genes found in at least one isolate.

### Statistical analysis

Either number and percent or median and interquartile range were calculated for descriptive analysis. Differences were tested using the Chi-square test or Wilcoxon rank-sum test. To analyze risk factors for the occurrence of ST117, we used a logistic regression model for multivariable analysis with patient age and sex, calendar year, type of ward, length of stay (LOS) at the time of specimen collection, and specimen collection site as possible confounders. Parameters with a significance of *p* ≤ 0.05 were entered into the model. All tests of significance were two-tailed, and *p* < 0.05 was considered statistically significant. All statistical analyses were performed using SPSS (IBM SPSS statistics, Somer, USA) and SAS (SAS Institute, Cary, USA).

### Ethics

The bacterial isolates were collected in the course of routine active surveillance and infection prevention control according to guidelines of National Healthcare Authorities. Personal data were anonymized and handled in compliance with the General Data Protection Regulation and medical-ethical guidelines of Charité - Universitätsmedizin Berlin for anonymized use of patient materials. Therefore, ethical approval by an institutional board was not necessary.

## Results

### Patient characteristics

All VREfm isolates from the years 2008, 2013, 2015 and 2018 were analyzed and came to a total of 120 cases. We investigated patient and isolate details, including age, sex, LOS, LOS at the time of specimen collection, type of ward, and site of specimen collection. In single quarters of each year, a variety of different wards were represented. The VREfm cases did not differ among calendar years with regard to age (in total median across all calendar years 66 years, IQR 53–75, *p* = 0.839), gender (in total male 52% and female 48%, *p* = 0.223), LOS (in total median 33 days, IQR 14–64%, *p* = 0.209), or the above mentioned ward type. Between 2008 and 2018, there was no difference in intensive care unit, hematology/oncology, surgery, or others (*p* = 0.945, *p* = 0.825, *p* = 0.867 and *p* = 0.729). In contrast, LOS at the time of specimen collection and site of specimen collection differed in various calendar years. In 2008, most of the samples were obtained as clinical cultures (*n* = 25, 83.3%), while in 2013 (*n* = 4, 13.3%), 2015 (*n* = 6, 20%), and 2018 (*n* = 7, 23.3%) screening cultures were more frequent. Urine accounted for the majority of clinical cultures. The LOS at the time of specimen collection decreased from 2008 (18.5 days; IQR 4–40) to 2018 (1.5 days; IQR 0–21) and is consequently of significance, *p* = 0.011.

### Characteristics of VREfm isolates

**Fig. 1:**
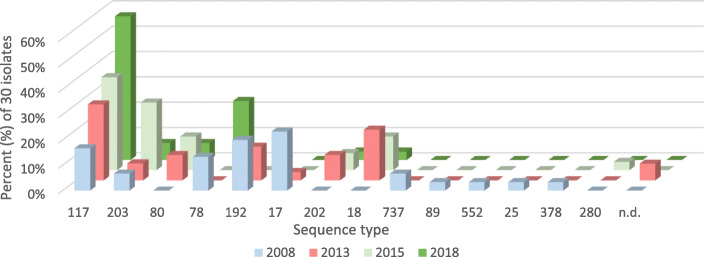
Frequency of different STs in percent for the period 2008 to 2018 with 30 isolates per year (n.d. denotes ST not defined, comprises different strains)

All 120 VREfm isolates were sequenced, with an average coverage ranging from 41- to 121-fold. The percentage of good targets based on the core genome ranged from 95.2 to 99.8% with an average of 99.0%. STs as well as CTs were determined for all strains. Regarding classification into ST, each consecutive year saw a higher percentage of isolates that were ST117, rising from 16.7% in 2008 to 56.7% in 2018 (*p* = 0.012). In total, 43 (35.8%) of the 120 *E. faecium* isolates were classified as ST117. In addition to ST117 strains, we detected strains assigned to ST203 (11.7%), ST80 (7.5%), ST78 (9.2%), ST192 (8.3%), ST17 (6.7%), and others (each ≤5%) (Fig. 1). While ST117, ST203 and ST80 were identified in all years, the number of isolates belonging to ST203 and ST80 increased until 2015 but decreased subsequently from 2015 to 2018. Beside the increase in ST117, we also observed a rise in isolates assigned to ST78 between 2015 and 2018 as well as a higher diversity of STs in 2008 than in 2018 (Fig. 1).

**Fig. 2:**
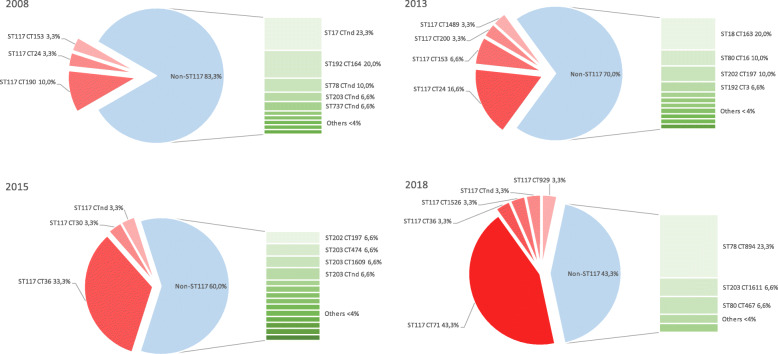
Increase in ST117 strains in 2008, 2013, 2015 and 2018. Pie chart: percentage of ST117 strains (marked in red) and non-ST117 strains (marked in light blue) based on 30 isolates per year, occurrence of different CTs within ST117 (marked in red); bar chart: occurrence of various CTs (marked green) within the non-ST117 group

When classifying the strains with a higher resolution into cgMLST, there was a clear shift of dominant CTs from CT164 in 2008 to CT71 in 2018. We did not find any CT71 isolates in 2008, 2013, or 2015 although there was a variety of other CTs such as CT24, CT36 and CT190 (Fig. 2). In contrast, 43% of isolates from 2018 were CT71 strains (13/30, *p* < 0.0001). Of all 120 isolates of the years studied, CT71 (11%) was the most common CT, followed by CT36 and CT162 (both 8%), and CT164 and CT894 (both 6%).

### Risk factors for the frequent occurrence of ST117 strains

Table 1 shows patient characteristics for ST117 and non-ST117 carriers in each year. Non-ST117 comprised all strains other than ST117, including those which could not be assigned to a known ST. Regarding ward type, 54% of all CT71 strains were collected in ICUs, which comprised five different ICUs located in different buildings across the city. Most samples were obtained from rectal swabs (*n* = 76, 63%), urine samples (*n* = 14, 12%) and stool samples (*n* = 9, 7.5%). Regarding the rectal swabs, there is a great increase from 2008 (*n* = 4) to 2013 (*n* = 26), whereas the number remained approximately constant in the following years (2015 *n* = 24 and 2018 *n* = 22). Urine samples accounted for 12% of the total number of samples, including 6 samples (20%) in 2008, 1 sample (3%) in 2013, 3 samples (10%) in 2015, and 4 samples (13%) in 2018. Stool samples only occurred in 2008 (n = 9) and no samples in 2013, 2015 or 2018. Most CT71 strains (n = 9; 70%) were obtained from rectal swabs.

**Table 1:** Patient characteristics among non-ST117 and ST117 carriers per year (2008-2018); patient characteristics of CT71 strains in 2018 (IQR: interquartile range; LOS: length of stay)

Year Total number of isolates	2008 *N* = 30		2013 *N* = 30		2015 *N* = 30		2018 *N* = 30		
Strain type No. (%)	Non-ST117 25 (83)	ST117 5 (17)	Non-ST117 21 (70)	ST117 9 (30)	Non-ST117 18 (60)	ST117 12 (40)	Non-ST117 13 (43)	ST117 17 (57)	CT71 13 (43)
**Age in years**, Median (IQR)	62 (46–72)	69 (56–76)	68 (57–76)	68 (53–71)	72.5 (54–74)	64 (47–72)	60 (54–66)	69 (52–77)	69 (52–75)
**Sex,** No. (%)									
Male	15 (60)	3 (60)	9 (43)	5 (56)	6 (33)	6 (50)	6 (46)	13 (77)	10 (77)
Female	10 (40)	2 (40)	12 (57)	4 (44)	12 (67)	6 (50)	7 (54)	4 (23)	3 (23)
**LOS in days**, Median (IQR)	53 (26–76)	23 (4–74)	32 (15–60)	47 (26–59)	31 (16–52)	34 (15–48)	14 (5–66)	16 (8–55)	40 (16–60)
**LOS specimen collection in days**, Median (IQR)	19 (6–37)	4 (4–42)	11 (2–24)	19 (11–26)	4.5 (1–12)	5 (1–18.5)	1 (0–42)	2 (0–12)	5 (1–21)
**Type ward,** No. (%)									
Intensive care unit	10 (40)	2 (40)	9 (43)	5 (56)	10 (56)	3 (25)	4 (30)	8 (47)	7 (54)
Hematology/oncology	9 (36)	1 (20)	8 (38)	3 (33)	4 (22)	7 (59)	5 (40)	3 (18)	3 (23)
Surgery	3 (12)	1 (20)	1 (5)	1 (11)	1 (5)	1 (8)	4 (30)	0 (0)	0 (0)
others	3 (12)	1 (20)	3 (14)	0 (0)	3 (17)	1 (8)	0 (0)	6 (35)	3 (23)
**Site of specimen collection**, No. (%)									
Screening cultures	4 (16)	1 (20)	19 (90)	7 (78)	13 (72)	11 (92)	12 (92)	11 (65)	9 (69)
Clinical cultures	21 (84)	4 (80)	2 (10)	2 (22)	5 (28)	1 (8)	1 (8)	6 (35)	4 (31)

### Multivariable risk factor analysis for ST117

The multivariable analysis supported a strong association of ST117 with the calendar year (Additional file 1: Table S1). Samples from 2018 were more than 9 times more likely to be type ST117 than in 2008 (OR 9.4, 95%CI 2.3–37.7, *p* = 0.002). A similar association was found for urine as specimen collection site and ST117 strains (OR 10.6, 95%CI 1.4–82.5, *p* = 0.024). CT71 was not an independent risk factor for ST117. Because CT71 did not appear until 2018, the calendar year could not be estimated in the model.

### Antimicrobial resistance, virulence factors and pangenome analysis of ST117 strains

Resistance genes and virulence factors for all 43 ST117 strains were identified using ResFinder, VirulenceFinder, VFDB, and CARD, all of which indicated various resistance genes and virulence factors (Additional file 2: Table S2). Resistance genes for macrolides, lincosamides, and streptogramin B (*msrC*, *erm(B)* and *efmA*) as well as for trimethoprim (*dfrF and dfrG*) and aminoglycosides (*aac(6′)-aph(2″)*, *aph(3′)-III*, *ant(6)-Ia and aac(6′)-Ii*) were detected in all ST117 strains. Moreover, genes for ciprofloxacin resistance (*gyrA, parC*) and ampicillin resistance (*pbp5*) were identified in all ST117 strains, which are intrinsic genes and expected to be present in all isolates. While almost all non-CT71 strains had several genes conferring resistance to aminoglycosides, we found only one such gene (*aac(6′)-Ii*) in CT71 strains, which is also an intrinsic gene. Some non-CT71 isolates featured resistances to chloramphenicol (*cat*) and tetracycline (*tet(M)*) which were absent in CT71 strains. Among all ST117 strains, 74% (32/43 isolates) displayed the *vanB* genotype and the *vanA* in 26% (11/43 isolates). There was a shift from *vanA* to *vanB* between 2008 and 2018, with 80% (4/5 isolates) and 67% (6/9 isolates) *vanA* in 2008 and 2013, to 100% (11/11 isolates) and 94% (16/17 isolates) *vanB* in 2015 and 2018, respectively (Additional file 3: Figure S1). All CT71 strains harbored *vanB*.

All ST117 strains carried the virulence factor *acm*, which encourages cell wall-anchored collagen adhesion and has characteristics typical of a microbial surface component recognizing adhesive matrix molecules (MSCRAMM) and *sgrA* to stimulate surface adhesion. Moreover, nearly all ST117 strains were characterized by the presence of *ecbA* (98%, 42/43 isolates), the *E. faecium* collagen binding protein A, as well as the enterococcal surface protein *esp* (95%, 41/43 isolates) to promote biofilm formation. The presence of a putative glycoside hydrolase *hylEfm* was detected in more than half of the ST117 strains (78%, 33/43 isolates). Occasionally, the virulence factor *scm* (14%, 6/43 isolates), the second collagen adhesin of *E. faecium*, occurred. All CT71 strains presented the same set of virulence factors: *acm*, *esp*, *hylEfm*, *ecbA*, and *sgrA* (30%, 13/43 isolates). This set of virulence factors was also detected in another 15 non-CT71 strains, a total of 28 isolates out of 43 (65%). These 15 strains belonged mainly to CT36 (21%, 9/43 isolates). The virulence factors of non-ST117 strains showed no striking difference from ST117 strains.

Regarding the alcohol tolerance assay, we tested five CT71 strains and one VREfm ATCC strain (ATCC 700221). At an isopropanol concentration of 23 and 60%, we could not detect any growth in any of the strains.

The phylogenetic tree with all ST117 strains revealed that the CT71 strains were phylogenetically separated from non-CT71 strains (Additional file 4: Figure S2). Additionally, a pangenome analysis was conducted with all ST117 strains to study the differences in gene content between CT71 strains and non-CT71 strains (*n* = 43). The isolates shared a core genome of 2166 genes (42 < = strains <=43), a soft-core genome of 130 genes (40 < = strains < 42), a shell genome of 1338 genes (6 < = strains < 40) and 1618 cloud genes (strains < 6). In summary, the core genome comprises 42% of the pangenome (5252 genes). The pangenome matrix (presence and absence of genes) revealed that the distribution of genes among strains was relatively similar. With regard to the accessory genome profile of the CT71 strains compared to the other CT strains, there seemed to be a set of genes more frequently present in CT71 strains than in the other strains (Additional file 5: Figure S3). Additionally, the hierarchical clustering based on presence and absence of genes clustered CT71 isolates distinctly from non-CT71 strains. The frequency chart shows that many genes were present in all genomes and that many genes were present only in single genomes, but we could not detect peaks for a set of isolates indicative of CT71 strains (*n* = 13) (Additional file 5: Figure S3).

## Discussion

This retrospective analysis revealed that the percentage of VREfm ST117 strains at Charité more than tripled between 2008 and 2018. When CT71 first appeared in 2018, it comprised more than 40% of all ST117 strains. In comparison, VREfm prevalence at Charité rose from 1.2% in 2016 to 1.4% in 2018. Thus, an overall increase of VRE may not be the only explanation for the rise of ST117/CT71.

Previous publications have also reported a dramatic increase of ST117. Liese et al. reported the frequent occurrence of ST117, ST80, ST17, and ST192 strains in outbreak analyses from a German university hospital between 2010 and 2016, with ST117 strains appearing frequently only at the end of 2015 and 2016 [22]. The German National Reference Centre for Staphylococci and Enterococci reported the same STs as Liese et.al. as well as an increase in ST117 in Germany in recent years [8]. An occurrence of ST117 strains was also observed in other European countries such as Denmark [23], Switzerland [24], Norway [25], the Netherlands [26, 27], and Spain [28].

In this study, different CTs of ST117 strains were identified; in 2018, however, CT71 clearly predominated. The analysis was comprised of non-outbreak isolates only, which were collected in different wards. Recent publications indicate the spread of CT71 throughout Germany in hospitals in different geographical regions without any presumed patient transfer [8, 29]. The recent predominance of ST117 CT71 both in outbreak and non-outbreak strains leads to a question: What could be facilitating the spread of this particular clonal lineage? Compared to other CTs among ST117 strains, we did not detect resistance or virulence traits in CT71 that could confer survival advantage. The detected resistance genes (*msrC*, *efmA, erm(B)*, *dfrG*, and *aac(6′)-Ii)* and virulence factors (*acm*, *esp*, *hylEfm*, *ecbA* and *sgrA*) of CT71 strains have already been reported in connection with high risk *Enterococcus* strains [23, 24, 30, 31]. The same set of virulence factors as in CT71 strains was also found in 15 other ST117 non-CT71 strains, mainly CT36, a CT frequently identified in 2015 (33%). So perhaps it is not a single trait that is responsible for the dominance and spread of specific strain types, but a combination of particular virulence factors.

Lee et al. has also reported the detection of the virulence factors *acm* and *sgrA* in connection with the predominance of an outbreak strain type ST173 [32]. Surprisingly, they identified fewer virulence factors in that outbreak strain than in the other strains. Falgenhauer et al. detected *acm*, *hylEfm*, and *esp* as well as the *efaAfm* gene in CT71 strains, the latter of which contributes to cell wall adherence [29]. CT71 strains were phylogenetically separated from non-CT71 strains, but the pangenome analysis did not reveal any explanation for the dominance of CT71 strains. Future research should address in-depth analysis of CT71-enriched pangenome genes, but is beyond the scope of this work.

The analysis of patient characteristics did not provide an explanation for the predominance of either ST117 or CT71 strains. Only the year of specimen collection and urine as the site of specimen collection increased the chance of finding ST117.

Even though urine as sample collection site is generally regarded as a clinical culture, the sampling site itself without any further clinical information did not necessarily prove that the patients had a urinary tract infection.

In contrast to most previous publications, we aimed to investigate isolates outside of reported outbreaks. Even though certain clones predominated in different years, continuous outbreak scenarios seem unlikely because the strains were found in three geographically distinct hospital buildings without an apparent epidemiological link between the patients. There may be ongoing inter-hospital spread, which would corroborate recent findings of Falgenhauer et.al., who also detected near-identical isolates (≤10 cgMLST alleles) of ST117 CT71 *vanB* clones in different hospitals across the Rhine-Main area of Germany [29]. Raven et al. also discussed the complexity of VREfm transmission chains, including both within and inter-hospital spread [33]. Inter-hospital spread would either require the direct movement of patients between the different hospitals or unrecognized vectors linking the strains.

Horizontal gene transfer may facilitate the spread of VREfm through the exchange of mobile genetic elements such as transposons or plasmids, or a crossover between chromosomal and plasmid DNA through insertion elements [34]. Zhou et al. have demonstrated that the detection of horizontal gene transfer is important in order to understand the complex transmission routes and outbreaks of VREfm [26]. Pinholt et al. have reported a clonal expansion based on a *vanA*-plasmid that was transferred via horizontal gene transfer to already existing hospital-adapted vancomycin-susceptible *E.faecium* and, thus, generated new VREfm [23]. The exchange of genomic material between VRE and VSE may be responsible for the dissemination of *vanB* resistance in Germany [35].

Besides the ST117 strain type there are also reports of other emerging dominant strains like ST796 [36] and ST203 [37]. Maybe there is an underlying selection leading to convergent evolution in different STs and CTs in different regions. Pidot et al. reported *E. faecium* strains with increased tolerance to handwash alcohols [10], whereas we could not detect a higher alcohol tolerance of CT71 strains.

Markwart et al. indicate specialist care hospitals and prevention and rehabilitation care centres as risk factors for a higher VRE proportion as well as patients older than 40 years [38]. These findings may be related to a larger number of patients with co-morbidities and older patients who are more frequently hospitalized and exposed to antibiotics during their lives. Regarding this study, non-ST117 and ST117 strains were mainly obtained from older patients (median > 60 years). Colonization pressure is an important factor in the spread of VRE by colonized patients [39], so it can be assumed that a higher colonization pressure of CT71 strains also leads to an increased spread of CT71 strains. Furthermore, the colonization period could be a crucial factor as VRE may colonize the gastrointestinal tract for several months [39]. Risk factors for a prolonged carriage of VRE are surgical interventions, antibiotic use during the hospitalization, dialysis and discharge to other health facility [40]. Perhaps the CT71 strains favor a longer colonization, due to a still unknown fact, and thus contribute to the spread. In general, we could not identify any specific risk factors for the acquisition of CT71 strains in this study.

The general increase of VREfm in Germany was accompanied by an increase in *vanB*-type strains [8] and by a shift from *vanA* to *vanB* genotype, which has been reported since 2015/2016 [22, 35]. We saw the same effect in our study, with an increase in *vanB* CT36 in 2015 and *vanB* CT71 in 2018.

Limitations of the study are the relatively small number of samples and the lack of information on patient factors both outside and inside of the hospital, such as comorbidities, antibiotic therapy, and admission to other health care institutions. In addition, the change in the numbers of active surveillance cultures for VRE and the days of LOS at specimen collection between 2008 and 2018 may have resulted in a systematic selection bias. The numbers of clinical and screening samples varied between the study years. Clinical cultures dominated in 2008, including isolates from patients with suspected or existing infection, and may therefore have more pathogenic properties. In 2013, 2015 and 2018 most samples were obtained from screening cultures. In general, there has been an increase in screening cultures throughout Germany. According to the German national nosocomial infection surveillance system (Krankenhaus-Infektions-Surveillance-System, KISS, https://www.nrz-hygiene.de/surveillance/kiss/) the percentage of ICUs with active surveillance screening for VRE increased from 50% in 2013 to 86% in 2018. Surveillance cultures and early screening (decreased LOS at specimen collection) as opposed to clinical cultures and screening later during the patients’ stay on the ward may reveal different strains.

A further limitation could be the databases that were used. Although we used two databases for the detection of virulence factors, there may be virulence factors that have not yet been reported. For example, higher colonization densities could be the result of additional genes encoding metabolic pathways in ST117 CT71 which are usually not listed in virulence databases.

## Conclusion

In conclusion, this retrospective analysis reports frequencies of specific VREfm strains in a German university hospital over time. An increase in ST117 strains from 2008 to 2018 was accompanied by a shift to *vanB* CT71 strains in 2018. We found neither specific virulence factors nor alterations in the patient mix to explain the increase of ST117 CT71. To date, it is not clear why ST117, and strain type ST117 CT71 in particular, predominates over other strains. In addition to epidemiological data, further studies to understand the complex spread of VREfm strain types need to take into account horizontal gene transfer in VREfm as well as potentially unrecognized vectors such as VSEfm or other bacterial lineages, and interactions with the intestinal microbiome.

## Supplementary information

**Additional file 1: Table S1.** Multivariable model with uni- and multivariable estimates (LOS: length of stay; ICU: intensive care unit; IQR: interquartile range; 95%CI: 95% confidence interval).

**Additional file 2: Table S2.** Resistance genes and virulence factors of 43 ST117 strains. Resistance genes: *msrC*: Macrolide, Lincosamide and Streptogramin B resistance; *efmA*, *erm(B)*: Macrolide resistance; *dfrF*, *dfrG*: Trimethoprim resistance; *aac(6′)-aph(2″), aph(3′)-III, ant(6)-Ia, aac(6′)-Ii*: Aminoglycoside resistance; *cat*: Chloramphenicol resistance; *tetM*: Tetracycline resistance; gyrA, parC: Ciprofloxacin resistance; pbp5: Ampicillin resistance. Virulence factors: *acm*: Cell wall-anchored collagen adhesin; *esp*: Enterococcal surface protein; *hylEfm*: Putative glycoside hydrolase; *ecbA*: *E. faecium* collagen binding protein A; *sgrA*: surface adhesion; *scm*: second collagen adhesin of *E. faecium.*

**Additional file 3: Figure S1.** Proportion of *vanA* and *vanB* genotype of ST117 strains from 2008 to 2018.

**Additional file 4: Figure S2.** Phylogenetic approximately maximum-likelihood tree with all ST117 strains (*n* = 43) created via FastTreeMP and visualized with Microreact, coloured by CT71 (*n* = 13).

**Additional file 5: Figure S3.** Results of pangenome analysis of all ST117 strains (n = 43) using roary_plots python script.

## Data Availability

All data generated or analyzed in the course of this study have been included in this published article and its supplementary information files.

## Abbreviations

cgMLST: Core genome multi locus sequence typing
Charité: Charité - Universitätsmedizin Berlin
CT: Cluster type
ICU: Intensive care unit
MLST: Multi locus sequence typing
ST: Sequence type
VRE: Vancomycin-resistant *Enterococcus*
VREfm: Vancomycin-resistant *Enterococcus faecium*
