# Supplementary material for: Increase of vancomycin-resistant Enterococcus faecium strain type ST117 CT71 at Charité - Universitätsmedizin Berlin, 2008 to 2018
Source: Antimicrob Resist Infect Control. 2020 Jul 16;9:109. doi: 10.1186/s13756-020-00754-1 (PMC7364619; doi:10.1186/s13756-020-00754-1)
Supplement: Supplementary file 1 — Additional file 1: Table S1. Multivariable model with uni- and multivariable estimates (LOS: length of stay; ICU: intensive care unit; IQR: interquartile range; 95%CI: 95% confidence interval). [file 13756_2020_754_MOESM1_ESM.docx]

**Table S1** Multivariable model with uni- and multivariable estimates (LOS: length of stay; ICU: intensive care unit; IQR: interquartile range; 95%CI: 95% confidence interval)

**a) Patients characteristics in ST117 and Non-ST117 strains**

| Parameter | Category | Total | ST117 |  |  |  |
| --- | --- | --- | --- | --- | --- | --- |
|  |  |  | No | Yes | %ST117 | p-value* |
|  | 0 | No.(%) or Median(IQR) | No.(%) or Median(IQR) | No.(%) or Median(IQR) | % |  |
| Strains, No.(%) | Total | 120 (100) | 77 (100) | 43 (100) | 35,8 |  |
| Year | 2008 | 30 (25) | 25 (32.5) | 5 (11.6) | 16,7 | 0,011 |
|  | 2013 | 30 (25) | 21 (27.3) | 9 (20.9) | 30,0 |  |
|  | 2015 | 30 (25) | 18 (23.4) | 12 (27.9) | 40,0 |  |
|  | 2018 | 30 (25) | 13 (16.9) | 17 (39.5) | 56,7 |  |
| Age (years), Median (IQR) |  | 66 (52.5-74.5) | 66 (53-73) | 65 (52-77) |  | 0,797 |
| Percentile Group of Age | 1 | 30 (25) | 19 (24.7) | 11 (25.6) | 36,7 | 0,222 |
|  | 2 | 28 (23.3) | 17 (22.1) | 11 (25.6) | 39,3 |  |
|  | 3 | 32 (26.7) | 25 (32.5) | 7 (16.3) | 21,9 |  |
|  | 4 | 30 (25) | 16 (20.8) | 14 (32.6) | 46,7 |  |
| Sex, No.(%) | Male | 63 (52.5) | 36 (46.8) | 27 (62.8) | 42,9 | 0,092 |
|  | Female | 57 (47.5) | 41 (53.2) | 16 (37.2) | 28,1 |  |
| LOS (days), Median (IQR) |  | 32.5 (13.5-63.5) | 32 (15-66) | 33 (9-59) |  | 0,407 |
| Percentile Group of LOS | 1 | 30 (25) | 17 (22.1) | 13 (30.2) | 43,3 | 0,390 |
|  | 2 | 30 (25) | 22 (28.6) | 8 (18.6) | 26,7 |  |
|  | 3 | 30 (25) | 17 (22.1) | 13 (30.2) | 43,3 |  |
|  | 4 | 30 (25) | 21 (27.3) | 9 (20.9) | 30,0 |  |
| LOS at the time of specimen collection (days), Median(IQR) |  | 9 (1-25) | 11 (1-28) | 5 (1-22) |  | 0,324 |
| Percentile Group of LOS specimen collection | 1 | 35 (29.2) | 20 (26) | 15 (34.9) | 42,9 | 0,767 |
|  | 2 | 26 (21.7) | 17 (22.1) | 9 (20.9) | 34,6 |  |
|  | 3 | 30 (25) | 20 (26) | 10 (23.3) | 33,3 |  |
|  | 4 | 29 (24.2) | 20 (26) | 9 (20.9) | 31,0 |  |
| Screening cultures, No.(%) | No | 42 (35) | 30 (39) | 12 (27.9) | 28,6 | 0,223 |
|  | Yes | 78 (65) | 47 (61) | 31 (72.1) | 39,7 |  |
| Material | Ascites sample | 1 (0.8) | 1 (1.3) | 0 (0) | 0,0 | 0,187 |
|  | Blood sample | 7 (5.8) | 4 (5.2) | 3 (7) | 42,9 |  |
|  | Bronchial lavage | 1 (0.8) | 1 (1.3) | 0 (0) | 0,0 |  |
|  | Tissue from hip joint | 1 (0.8) | 1 (1.3) | 0 (0) | 0,0 |  |
|  | Intraoperative swab | 1 (0.8) | 0 (0) | 1 (2.3) | 100,0 |  |
|  | Midstream urine | 7 (5.8) | 5 (6.5) | 2 (4.7) | 28,6 |  |
|  | Nasal throat swab | 1 (0.8) | 1 (1.3) | 0 (0) | 0,0 |  |
|  | Renal fistula catheter urine | 1 (0.8) | 1 (1.3) | 0 (0) | 0,0 |  |
|  | Throat swab | 1 (0.8) | 0 (0) | 1 (2.3) | 100,0 |  |
|  | Rectal swab | 76 (63.3) | 46 (59.7) | 30 (69.8) | 39,5 |  |
|  | Bile secretion | 1 (0.8) | 1 (1.3) | 0 (0) | 0,0 |  |
|  | Stool sample | 9 (7.5) | 8 (10.4) | 1 (2.3) | 11,1 |  |
|  | Urine sample | 7 (5.8) | 2 (2.6) | 5 (11.6) | 71,4 |  |
|  | Wound swab | 6 (5) | 6 (7.8) | 0 (0) | 0,0 |  |
| ICU, No.(%) | No | 69 (57.5) | 44 (57.1) | 25 (58.1) | 36,2 | 0,916 |
|  | Yes | 51 (42.5) | 33 (42.9) | 18 (41.9) | 35,3 |  |
| Hematology/Oncology (Non ICU), No.(%) | No | 80 (66.7) | 51 (66.2) | 29 (67.4) | 36,3 | 0,893 |
|  | Yes | 40 (33.3) | 26 (33.8) | 14 (32.6) | 35,0 |  |
| Surgery (Non ICU), No.(%) | No | 108 (90) | 68 (88.3) | 40 (93) | 37,0 | 0,409 |
|  | Yes | 12 (10) | 9 (11.7) | 3 (7) | 25,0 |  |
| Other ward (Non ICU), No.(%) | No | 103 (85.8) | 68 (88.3) | 35 (81.4) | 34,0 | 0,297 |
|  | Yes | 17 (14.2) | 9 (11.7) | 8 (18.6) | 47,1 |  |
| Strain ST117, No.(%) | No | 77 (64.2) | 77 (100) | 0 (0) | 0,0 |  |
|  | Yes | 43 (35.8) | 0 (0) | 43 (100) | 100,0 |  |
| Strain CT71, No.(%) | No | 107 (89.2) | 77 (100) | 30 (69.8) | 28,0 | <.001 |
|  | Yes | 13 (10.8) | 0 (0) | 13 (30.2) | 100,0 |  |
| Year | 2008 | 30 (25) | 25 (32.5) | 5 (11.6) | 16,7 | 0,011 |
|  | 2013 | 30 (25) | 21 (27.3) | 9 (20.9) | 30,0 |  |
|  | 2015 | 30 (25) | 18 (23.4) | 12 (27.9) | 40,0 |  |
|  | 2018 | 30 (25) | 13 (16.9) | 17 (39.5) | 56,7 |  |
| Ascites sample | No | 119 (99.2) | 76 (98.7) | 43 (100) | 36,1 | 0,453 |
|  | Yes | 1 (0.8) | 1 (1.3) | 0 (0) | 0,0 |  |
| Blood sample | No | 113 (94.2) | 73 (94.8) | 40 (93) | 35,4 | 0,690 |
|  | Yes | 7 (5.8) | 4 (5.2) | 3 (7) | 42,9 |  |
| Bronchial lavage | No | 119 (99.2) | 76 (98.7) | 43 (100) | 36,1 | 0,453 |
|  | Yes | 1 (0.8) | 1 (1.3) | 0 (0) | 0,0 |  |
| Tissue from hip joint | No | 119 (99.2) | 76 (98.7) | 43 (100) | 36,1 | 0,453 |
|  | Yes | 1 (0.8) | 1 (1.3) | 0 (0) | 0,0 |  |
| Midstream urine | No | 113 (94.2) | 72 (93.5) | 41 (95.3) | 36,3 | 0,680 |
|  | Yes | 7 (5.8) | 5 (6.5) | 2 (4.7) | 28,6 |  |
| Nasal throat swab | No | 119 (99.2) | 76 (98.7) | 43 (100) | 36,1 | 0,453 |
|  | Yes | 1 (0.8) | 1 (1.3) | 0 (0) | 0,0 |  |
| Renal fistula catheter urine | No | 119 (99.2) | 76 (98.7) | 43 (100) | 36,1 | 0,453 |
|  | Yes | 1 (0.8) | 1 (1.3) | 0 (0) | 0,0 |  |
| Throat swab | No | 119 (99.2) | 77 (100) | 42 (97.7) | 35,3 | 0,179 |
|  | Yes | 1 (0.8) | 0 (0) | 1 (2.3) | 100,0 |  |
| Rectal swab | No | 44 (36.7) | 31 (40.3) | 13 (30.2) | 29,5 | 0,274 |
|  | Yes | 76 (63.3) | 46 (59.7) | 30 (69.8) | 39,5 |  |
| Bile secretion | No | 119 (99.2) | 76 (98.7) | 43 (100) | 36,1 | 0,453 |
|  | Yes | 1 (0.8) | 1 (1.3) | 0 (0) | 0,0 |  |
| Stool sample | No | 111 (92.5) | 69 (89.6) | 42 (97.7) | 37,8 | 0,108 |
|  | Yes | 9 (7.5) | 8 (10.4) | 1 (2.3) | 11,1 |  |
| Urine sample | No | 113 (94.2) | 75 (97.4) | 38 (88.4) | 33,6 | 0,043 |
|  | Yes | 7 (5.8) | 2 (2.6) | 5 (11.6) | 71,4 |  |
| Wound swab | No | 114 (95) | 71 (92.2) | 43 (100) | 37,7 | 0,060 |
|  | Yes | 6 (5) | 6 (7.8) | 0 (0) | 0,0 |  |
| Intraoperative swab | No | 119 (99.2) | 77 (100) | 42 (97.7) | 35,3 | 0,179 |
|  | Yes | 1 (0.8) | 0 (0) | 1 (2.3) | 100,0 |  |
| *p-values, chi-square or Wilcoxon rank sum test | |  |  |  |  |  |

**b) Multivariable risk factor analysis for ST117**

| **Parameter** | **Category** | **OR** | **95%CI** | **P-value** |
| --- | --- | --- | --- | --- |
| **Year** | 2008 | 1=reference |  | 0.014 |
|  | 2013 | 3.60 | 0.86-15.13 | 0.080 |
|  | 2015 | 5.60 | 1.37-22.92 | 0.017 |
|  | 2018 | 9.36 | 2.33-37.64 | 0.002 |
| **Material** | Urine | 10.62 | 1.37-82.52 | 0.024 |
