# Supplementary material for: Increase of vancomycin-resistant Enterococcus faecium strain type ST117 CT71 at Charité - Universitätsmedizin Berlin, 2008 to 2018
Source: Antimicrob Resist Infect Control. 2020 Jul 16;9:109. doi: 10.1186/s13756-020-00754-1 (PMC7364619; doi:10.1186/s13756-020-00754-1)
Supplement: Supplementary file 2 — Additional file 2: Table S2. Resistance genes and virulence factors of 43 ST117 strains. Resistance genes: msrC: Macrolide, Lincosamide and Streptogramin B resistance; efmA, erm(B): Macrolide resistance; dfrF, dfrG: Trimethoprim resistance; aac(6′)-aph(2″), aph(3′)-III, ant(6)-Ia, aac(6′)-Ii: Aminoglycoside resistance; cat: Chloramphenicol resistance; tetM: Tetracycline resistance; gyrA, parC: Ciprofloxacin resistance; pbp5: Ampicillin resistance. Virulence factors: acm: Cell wall-anchored collagen adhesin; esp: Enterococcal surface protein; hylEfm: Putative glycoside hydrolase; ecbA: E. faecium collagen binding protein A; sgrA: surface adhesion; scm: second collagen adhesin of E. faecium. [file 13756_2020_754_MOESM2_ESM.docx]

**Table S2** Resistance genes and virulence factors of 43 ST117 strains (n.d. denotes CT not defined)

Resistance genes: *msrC*: Macrolide, Lincosamide and Streptogramin B resistance; *efmA*, *erm(B)*: Macrolide resistance; *dfrF*, *dfrG*: Trimethoprim resistance; *aac(6')-aph(2''), aph(3')-III, ant(6)-Ia, aac(6')-Ii*: Aminoglycoside resistance; *cat*: Chloramphenicol resistance; *tetM*: Tetracycline resistance; *gyrA, parC*: Ciprofloxacin resistance; *pbp5*: Ampicillin resistance;

Virulence factors: *acm*: Cell wall-anchored collagen adhesin; *esp*: Enterococcal surface protein; *hylEfm*: Putative glycoside hydrolase ; *ecbA*: E. faecium collagen binding protein A; *sgrA*: surface adhesion; *scm*: second collagen adhesin of E. faecium

| **Nr.** | **Strain** | **ST** | **CT** | **Year** | **Van** | **Resistance genes** | **Virulence factors** |
| --- | --- | --- | --- | --- | --- | --- | --- |
| **1** | VRE5538 | ST117 | CT71 | 2018 | B | *msrC, efmA, erm(B), dfrG, aac(6')-Ii, gyrA, parC, pbp5* | *acm, esp, hylEfm, ecbA, sgrA* |
| **2** | VRE5550 | ST117 | CT71 | 2018 | B | *msrC, efmA, erm(B), dfrG, aac(6')-Ii, gyrA, parC, pbp5* | *acm, esp, hylEfm, ecbA, sgrA* |
| **3** | VRE6039 | ST117 | CT71 | 2018 | B | *msrC, efmA, erm(B), dfrG, aac(6')-Ii, gyrA, parC, pbp5* | *acm, esp, hylEfm, ecbA, sgrA* |
| **4** | VRE6040 | ST117 | CT71 | 2018 | B | *msrC, efmA, erm(B), dfrG, aac(6')-Ii, gyrA, parC, pbp5* | *acm, esp, hylEfm, ecbA, sgrA* |
| **5** | VRE6052 | ST117 | CT71 | 2018 | B | *msrC, efmA, erm(B), dfrG, aac(6')-Ii, gyrA, parC, pbp5* | *acm, esp, hylEfm, ecbA, sgrA* |
| **6** | VRE6410 | ST117 | CT71 | 2018 | B | *msrC, efmA, erm(B), dfrG, aac(6')-Ii, gyrA, parC, pbp5* | *acm, esp, hylEfm, ecbA, sgrA* |
| **7** | VRE6416 | ST117 | CT71 | 2018 | B | *msrC, efmA, erm(B), dfrG, aac(6')-Ii, gyrA, parC, pbp5* | *acm, esp, hylEfm, ecbA, sgrA* |
| **8** | VRE6417 | ST117 | CT71 | 2018 | B | *msrC, efmA, erm(B), dfrG, aac(6')-Ii, gyrA, parC, pbp5* | *acm, esp, hylEfm, ecbA, sgrA* |
| **9** | VRE6425 | ST117 | CT71 | 2018 | B | *msrC, efmA, erm(B), dfrG, aac(6')-Ii, gyrA, parC, pbp5* | *acm, esp, hylEfm, ecbA, sgrA* |
| **10** | V106 | ST117 | CT71 | 2018 | B | *msrC, efmA, erm(B), dfrG, aac(6')-Ii, gyrA, parC, pbp5* | *acm, esp, hylEfm, ecbA, sgrA* |
| **11** | V108 | ST117 | CT71 | 2018 | B | *msrC, efmA, erm(B), dfrG, aac(6')-Ii, gyrA, parC, pbp5* | *acm, esp, hylEfm, ecbA, sgrA* |
| **12** | V110 | ST117 | CT71 | 2018 | B | *msrC, efmA, erm(B), dfrG, aac(6')-Ii, gyrA, parC, pbp5* | *acm, esp, hylEfm, ecbA, sgrA* |
| **13** | V117 | ST117 | CT71 | 2018 | B | *msrC, efmA, erm(B), dfrG, aac(6')-Ii, gyrA, parC, pbp5* | *acm, esp, hylEfm, ecbA, sgrA* |
| **14** | VRE41 | ST117 | CT190 | 2008 | A | *msrC, efmA, erm(B), dfrF, aac(6')-aph(2''), aph(3')-III, ant(6)-Ia, aac(6')-Ii, gyrA, parC, pbp5* | *acm, esp, ecbA, sgrA* |
| **15** | VRE106 | ST117 | CT190 | 2008 | B | *msrC, efmA, erm(B), dfrF, dfrG, aac(6')-aph(2''), aph(3')-III, ant(6)-Ia, aac(6')-Ii, gyrA, parC, pbp5* | *acm, ecbA, sgrA, scm* |
| **16** | VRE136 | ST117 | CT190 | 2008 | A | *msrC, efmA, erm(B), dfrF, aac(6')-aph(2''), aph(3')-III, ant(6)-Ia, aac(6')-Ii, gyrA, parC, pbp5* | *acm, esp, hylEfm, ecbA, sgrA* |
| **17** | VRE137 | ST117 | CT153 | 2008 | A | *msrC, erm(B), dfrF, dfrG, aac(6')-aph(2''), aph(3')-III, ant(6)-Ia, aac(6')-Ii, cat; gyrA, parC, pbp5* | *acm, esp, ecbA, sgrA* |
| **18** | VRE1305 | ST117 | CT153 | 2013 | A | *msrC, erm(B), dfrF, dfrG, aac(6')-aph(2''), aph(3')-III, ant(6)-Ia, aac(6')-Ii, cat, gyrA, parC, pbp5* | *acm, esp, ecbA, sgrA* |
| **19** | VRE1308 | ST117 | CT153 | 2013 | A | *msrC, erm(B), dfrF, dfrG, aac(6')-aph(2''), aph(3')-III, ant(6)-Ia, aac(6')-Ii, cat, gyrA, parC, pbp5* | *acm, esp, ecbA, sgrA, scm* |
| **20** | VRE2611 | ST117 | CT36 | 2015 | B | *msrC, efmA, dfrG, aac(6')-aph(2''), aph(3')-III, ant(6)-Ia, aac(6')-Ii, gyrA, parC, pbp5* | *acm, esp, hylEfm, ecbA, sgrA* |
| **21** | VRE2617 | ST117 | CT36 | 2015 | B | *msrC, efmA, erm(B), dfrG, aac(6')-aph(2''), aph(3')-III, ant(6)-Ia, aac(6')-Ii, gyrA, parC, pbp5* | *acm, esp, hylEfm, ecbA, sgrA* |
| **22** | VRE2618 | ST117 | CT36 | 2015 | B | *msrC, efmA, erm(B), dfrG, aac(6')-aph(2''), aph(3')-III, ant(6)-Ia, aac(6')-Ii, gyrA, parC, pbp5* | *acm, esp, hylEfm, ecbA, sgrA* |
| **23** | VRE2619 | ST117 | CT36 | 2015 | B | *msrC, efmA, erm(B), dfrG, aph(3')-III, ant(6)-Ia, aac(6')-Ii, gyrA, parC, pbp5* | *acm, esp, hylEfm, ecbA, sgrA* |
| **24** | VRE2847 | ST117 | CT36 | 2015 | B | *msrC, efmA, erm(B), dfrG,* *aac(6')-aph(2''), aph(3')-III, ant(6)-Ia, aac(6')-Ii, gyrA, parC, pbp5* | *acm, esp, hylEfm, ecbA, sgrA* |
| **25** | VRE3076 | ST117 | CT36 | 2015 | B | *msrC, efmA, erm(B), dfrG, aac(6')-aph(2''), aph(3')-III, ant(6)-Ia, aac(6')-Ii, gyrA, parC, pbp5* | *acm, esp, hylEfm, ecbA, sgrA, scm* |
| **26** | VRE3077 | ST117 | CT36 | 2015 | B | *msrC, efmA, erm(B), dfrG, aph(3')-III, ant(6)-Ia, aac(6')-Ii, gyrA, parC, pbp5* | *acm, esp, hylEfm, ecbA, sgrA* |
| **27** | VRE3085 | ST117 | CT36 | 2015 | B | *msrC, efmA, erm(B), dfrG, aph(3')-III, ant(6)-Ia, aac(6')-Ii, gyrA, parC, pbp5* | *acm, esp, hylEfm, ecbA, sgrA* |
| **28** | VRE3278 | ST117 | CT36 | 2015 | B | *msrC, efmA, erm(B), dfrG, aph(3')-III, ant(6)-Ia, aac(6')-Ii, gyrA, parC, pbp5* | *acm, esp, hylEfm, ecbA, sgrA, scm* |
| **29** | VRE6041 | ST117 | CT36 | 2018 | B | *msrC, efmA, erm(B), dfrG, aph(3')-III, ant(6)-Ia, aac(6')-Ii , gyrA, parC, pbp5* | *acm, esp, hylEfm, ecbA, sgrA* |
| **30** | VRE3284 | ST117 | CT36 | 2015 | B | *msrC, efmA, erm(B), dfrG, aac(6')-aph(2''), aph(3')-III, ant(6)-Ia, aac(6')-Ii, gyrA, parC, pbp5* | *acm, esp, hylEfm, ecbA, sgrA* |
| **31** | VRE62 | ST117 | CT24 | 2008 | A | *msrC, efmA, erm(B), dfrF, dfrG, aac(6')-aph(2''), aac(6')-Ii, cat, gyrA, parC, pbp5* | *acm, esp, ecbA, sgrA* |
| **32** | VRE1696 | ST117 | CT24 | 2013 | B | *msrC, efmA, erm(B), dfrF, dfrG, aph(3')-III, ant(6)-Ia, aac(6')-Ii, gyrA, parC, pbp5* | *acm, esp, ecbA, sgrA* |
| **33** | VRE1693 | ST117 | CT24 | 2013 | A | *msrC, efmA erm(B), dfrF, dfrG, aac(6')-aph(2''), aac(6')-Ii, cat, gyrA, parC, pbp5* | *acm, esp, ecbA, sgrA* |
| **34** | VRE1703 | ST117 | CT24 | 2013 | B | *msrC, efmA, erm(B), dfrF, dfrG, aph(3')-III, ant(6)-Ia, aac(6')-Ii, gyrA, parC, pbp5* | *acm, esp, ecbA, sgrA* |
| **35** | VRE1827 | ST117 | CT24 | 2013 | A | *msrC, erm(B), dfrF, dfrG, aac(6')-aph(2''), ant(6)-Ia, aac(6')-Ii, cat, gyrA, parC, pbp5* | *acm, esp, ecbA, sgrA* |
| **36** | VRE1830 | ST117 | CT24 | 2013 | A | *msrC, erm(B), dfrF, dfrG, aac(6')-aph(2''), aph(3')-III, ant(6)-Ia, aac(6')-Ii, cat, gyrA, parC, pbp5* | *acm, esp, ecbA, sgrA, scm* |
| **37** | V111 | ST117 | CT929 | 2018 | A | *msrC, efmA, erm(B), dfrG, aac(6')-aph(2''), aph(3')-III, ant(6)-Ia, aac(6')-Ii, gyrA, parC, pbp5* | *acm, esp, hylEfm, ecbA, sgrA* |
| **38** | VRE1313 | ST117 | CT1489 | 2013 | B | *msrC, efmA, erm(B), dfrF, dfrG, aac(6')-aph(2''), aph(3')-III, ant(6)-Ia, aac(6')-Ii, cat, tetM, gyrA, parC, pbp5* | *acm, hylEfm, sgrA* |
| **39** | VRE5560 | ST117 | CT1526 | 2018 | B | *msrC, efmA, erm(B), dfrG, aph(3')-III, ant(6)-Ia, aac(6')-Ii, gyrA, parC, pbp5* | *acm, esp, hylEfm, ecbA, sgrA* |
| **40** | VRE1484 | ST117 | CT200 | 2013 | A | *msrC, erm(B), dfrF, dfrG, aac(6')-aph(2''), aph(3')-III, ant(6)-Ia, aac(6')-Ii, cat, gyrA, parC, pbp5* | *acm, esp, hylEfm, ecbA, sgrA, scm* |
| **41** | VRE2845 | ST117 | CT30 | 2015 | B | *msrC, erm(B), efmA, dfrF, dfrG, aph(3')-III, ant(6)-Ia, aac(6')-Ii, gyrA, parC, pbp5* | *acm, esp, hylEfm, ecbA, sgrA* |
| **42** | VRE2849 | ST117 | n.d. | 2015 | B | *msrC, efmA, erm(B), dfrG, aph(3')-III, ant(6)-Ia, aac(6')-Ii, tetM, gyrA, parC, pbp5* | *acm, esp, hylEfm, ecbA, sgrA* |
| **43** | VRE5551 | ST117 | n.d. | 2018 | B | *msrC, efmA, erm(B), dfrG, aac(6')-Ii, tetM, gyrA, parC, pbp5* | *acm, esp, hylEfm, ecbA, sgrA* |
