# Supplementary figures and images for: Increase of vancomycin-resistant Enterococcus faecium strain type ST117 CT71 at Charité - Universitätsmedizin Berlin, 2008 to 2018
Source: Antimicrob Resist Infect Control. 2020 Jul 16;9:109. doi: 10.1186/s13756-020-00754-1 (PMC7364619; doi:10.1186/s13756-020-00754-1)

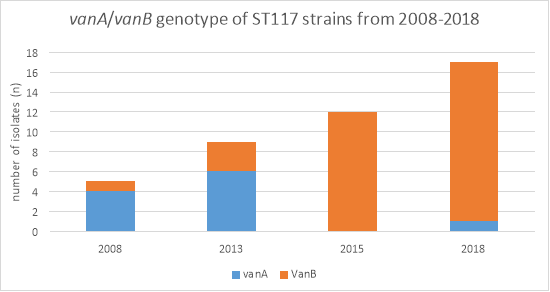


**Fig. S1** Proportion of *vanA* and *vanB* genotype of ST117 strains from 2008-2018

Supplement: Supplementary file 3 — Additional file 3: Figure S1. Proportion of vanA and vanB genotype of ST117 strains from 2008 to 2018. [file 13756_2020_754_MOESM3_ESM.docx]
