# Supplementary material for: Increase of vancomycin-resistant Enterococcus faecium strain type ST117 CT71 at Charité - Universitätsmedizin Berlin, 2008 to 2018
Source: Antimicrob Resist Infect Control. 2020 Jul 16;9:109. doi: 10.1186/s13756-020-00754-1 (PMC7364619; doi:10.1186/s13756-020-00754-1)
Supplement: Supplementary file 4 — Additional file 4: Figure S2. Phylogenetic approximately maximum-likelihood tree with all ST117 strains (n = 43) created via FastTreeMP and visualized with Microreact, coloured by CT71 (n = 13). [file 13756_2020_754_MOESM4_ESM.docx]

**
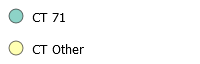
**
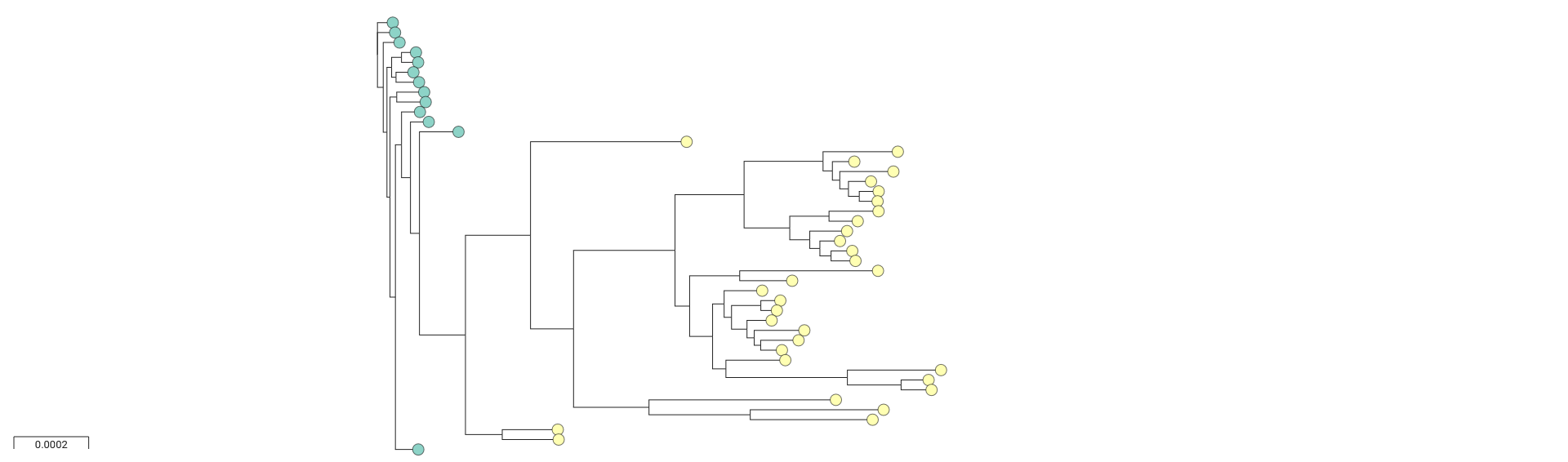


**Fig. S2** Phlyogenetic approximately maximum-likelihood tree with all ST117 strains (n=43) created via FastTreeMP and visualized with Microreact, coloured by CT71 (n=13)
