# Supplementary material for: Increase of vancomycin-resistant Enterococcus faecium strain type ST117 CT71 at Charité - Universitätsmedizin Berlin, 2008 to 2018
Source: Antimicrob Resist Infect Control. 2020 Jul 16;9:109. doi: 10.1186/s13756-020-00754-1 (PMC7364619; doi:10.1186/s13756-020-00754-1)
Supplement: Supplementary file 5 — Additional file 5: Figure S3. Results of pangenome analysis of all ST117 strains (n = 43) using roary_plots python script. [file 13756_2020_754_MOESM5_ESM.docx]

**a)**


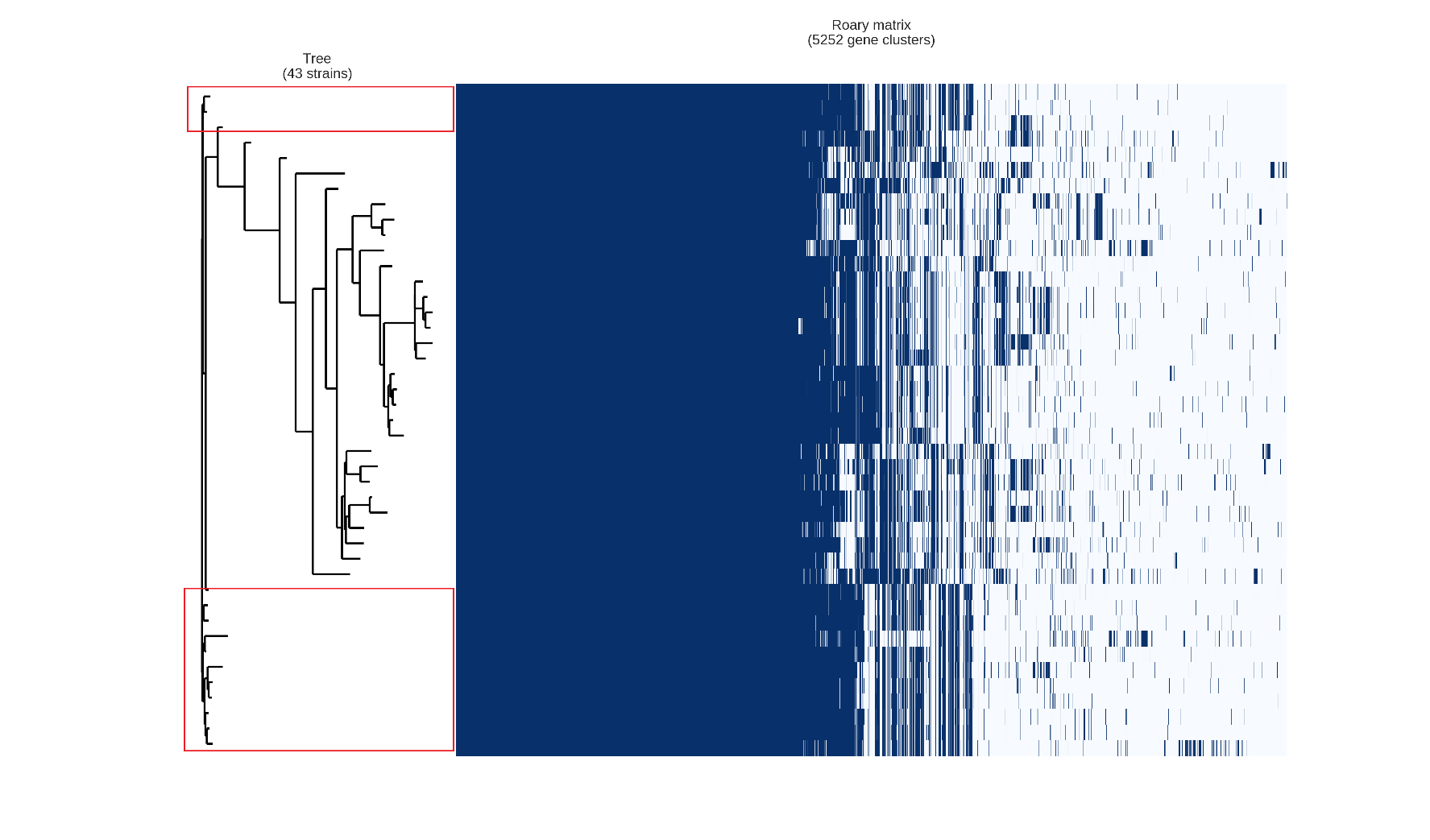


**b)**


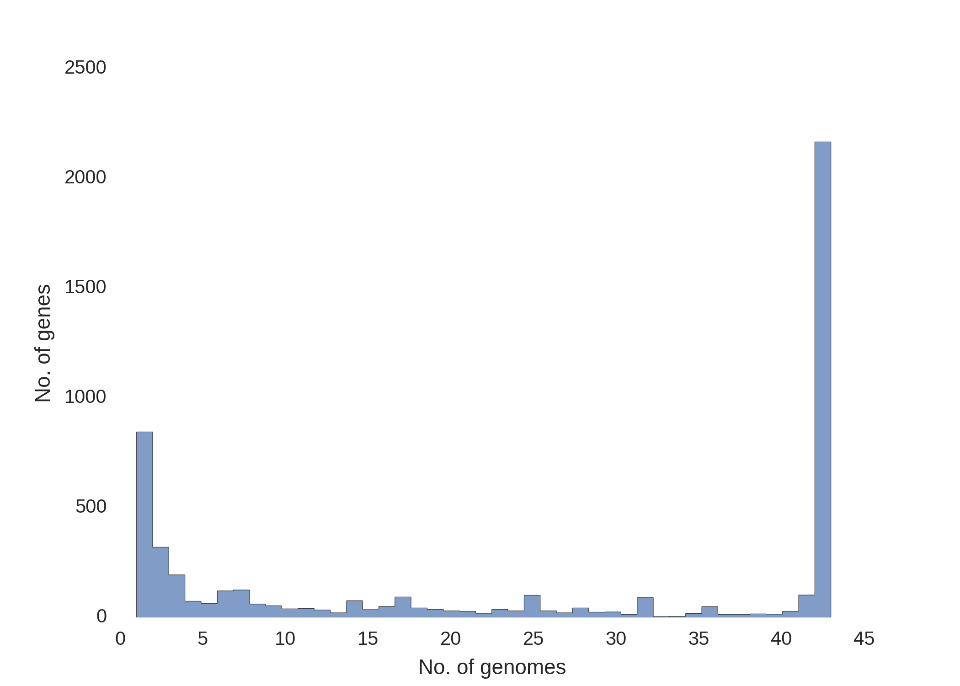


**Fig. S3** Results of pangenome analysis of all ST117 strains (n=43) using roary_plots python script
a) Pangenome matrix (presence and absence of genes) of the individual strains, CT71 strains are marked in red
b) Frequency chart with the number of genes present in a given number of genomes; Left side of the x-axis shows the genes present in a single genome, right side of the x-axis shows the genes present in all genomes (n=43)
